# *Plasmodium falciparum*, but not *P. vivax*, can induce erythrocytic apoptosis

**DOI:** 10.1186/s13071-014-0484-8

**Published:** 2014-10-18

**Authors:** Paulo Renato Rivas Totino, Aline das Dores Magalhães, Eliana Brasil Alves, Monica Regina Farias Costa, Marcus Vinícius Guimarães de Lacerda, Cláudio Tadeu Daniel-Ribeiro, Maria de Fátima Ferreira-da-Cruz

**Affiliations:** Laboratório de Pesquisas em Malária, Instituto Oswaldo Cruz, Fiocruz, Rio de Janeiro, Brazil; Centro de Pesquisa, Diagnóstico e Treinamento em Malária, Secretaria de Vigilância em Saúde, Ministério da Saúde, Brazil; Fundação de Hematologia e Hemoterapia do Amazonas, Manaus, Brazil; Fundação de Medicina Tropical Dr. Heitor Vieira Dourado, Manaus, Brazil

## Abstract

**Background:**

Apoptosis can occur in red blood cells (RBC) and seems to be involved in hematologic disorders related to many diseases. In malaria it is known that parasitized RBC (pRBC) is involved in the development of anemia and thrombosis; however, non-parasitized RBC (nRBC) apoptosis could amplify these malaria-associated hematologic events. In fact, in experimental malaria, increased levels of apoptosis were observed in nRBC during lethal *Plasmodium yoelii* 17XL infection, but in human malaria erythrocytic apoptosis has never been studied. The present study was performed to investigate if nRBC apoptosis also occurs in *P. vivax* and *P. falciparum* infections.

**Findings:**

Apoptosis of nRBC was evaluated in blood samples of *P. vivax* malaria patients and clinically healthly individuals living in Manaus, Brazil, both *ex vivo* and after incubation of RBC for 24 h. Additionally, the capacity of plasma from *P. vivax* or *P. falciparum* patients was tested for induction of *in vitro* apoptosis of normal RBC from a clinically healthy individual living in a non-endemic malaria region. Apoptosis was detected by flow cytometry using annexin V staining. In contrast to experimental malaria that significantly increased the levels of apoptotic nRBC both *ex-vivo* and after 24 h of incubation, no significant alteration on apoptotic nRBC rates was detected in *P. vivax* infected patients when compared with non-infected control individuals. Similar results were observed when plasma of these *P. vivax* patients was incubated with normal RBC. Conversely, plasma from *P. falciparum*-infected subjects induced significant apoptosis of these cells.

**Conclusions:**

Apoptosis of normal RBC can be induced by plasma from individuals with *P. falciparum* (but not with *P. vivax*) malaria. This finding could reflect the existence of erythrocytic apoptosis during infection that could contribute to the pathogenesis of hematological and vascular complications associated with *falciparum* malaria.

## Findings

It is currently known that the physiological processes of apoptotic cell death are not restricted to nucleated cells, occurring also in cells lacking a nucleus and organelles, such as the red blood cells (RBC) [1]. Similar to apoptosis of nucleated cells, erythrocytic apoptosis is triggered by different endogenous and exogenous stimuli and is characterized by many cellular changes leading to cell elimination [1,2]. These changes include exposure of phosphatidylserine (PS) on the cell surface, which drives dying cells to degradation by phagocytosis [3] or, alternatively, mediates cell adhesion on endothelium [4]. It is believed, therefore, that erythrocytic apoptosis could participate in the pathogenesis of clinical disorders in which enhanced levels of apoptotic RBC are a common feature, such as iron and glucose-6-phosphate dehydrogenase-(G6PD) deficiency, diabetes, renal insufficiency, hemolytic uremic syndrome, sickle-cell disease, sepsis and mycoplasma infection [2].

In malaria, pRBC apoptosis was detected in experimental *P. yoelii* 17XL [5] and *P. berghei* ANKA [6] rodent malaria as well as *in vitro P. falciparum* culture [7], and this process could contribute to parasite clearance. However, an increase in the levels of apoptotic non-parasitized RBC (nRBC) was further evidenced in *P. yoelii* infection, pointing to the putative role of erythrocytic apoptosis in the pathogenesis of malaria [8]. Since apoptotic nRBC has not yet been assessed in human malaria infections, the present study attempted to address this issue in Brazilian malarious patients.

For this, the levels of nRBC apoptosis were examined in peripheral blood of 20 malarious patients infected by *P. vivax* – the species that accounts annually for around 82% of malaria cases in Brazil [9,10]. Patients presenting positive by thick blood smear were recruited at the Tropical Medicine Foundation Dr. Heitor Vieira Dourado (Manaus, Amazonas, Brazil) and, then, venous blood samples were collected in EDTA tubes. Plasma and RBC were separated by centrifugation at 350 *g* for 10 min; plasma was stored in liquid nitrogen and RBC were used for apoptosis assays. Blood samples from 10 clinically healthy individuals living in the same area and presenting as negative by thick blood smear and no history of previous malaria episodes were used as controls. During the blood collection no cases of *P. falcipar*um malaria or complicated *P. vivax* malaria attended the Tropical Medicine Foundation. The study was approved by the Tropical Medicine Foundation Ethical Committee.

Parasitemia was estimated by examination of thick blood smears using a semi-quantitative method, as described previously [11]. As shown in Table 1, the majorly of *P. vivax* patients (n = 11) presented parasitemia ranging from 501 to 10,000 parasites/μL. In two patients parasitemia was between 301 and 500 parasites/μL and in seven it was less than 300.

**Table 1:** **Semi-quantitative parasitemia in** ***P. vivax*** **and** ***P. falciparum*** **patients, according to Lança** ***et al*** **., 2012** [11]

	**Semi-quantitative parasitemia (parasites/μL)**			
	**<+**	**+**	**++**	**+++**
	**(≤300)**	**(301–500)**	**(501–10,000)**	**(10,001-100,000)**
***P. vivax*** **(n = 20)**	**07**	**02**	**11**	**---**
***P. falciparum*** **(n = 10)**	**---**	**---**	**08**	**02**
**n = number of patients**				

Apoptosis was assayed both *ex vivo* and after RBC incubation for 24 h at 37°C at a hematocrit of 0.5% in Ringer solution containing (in mM) 125 NaCl, 5 KCl, 1 MgSO_4_, 32 N-2-hydroxyethylpiperazine-N-2-ethanesulfonic acid (HEPES), 5 glucose, and 1 CaCl_2_ (pH 7.4). Apoptotic nRBC was detected through flow cytometry using Syto 16 and annexin V-PE double staining, as performed in our previous studies with *P. yoelii* 17XL [5,8]. However, in contrast to experimental malaria, that significantly increased the levels of apoptotic nRBC both *ex-vivo* and after 24 h incubation, no significant alteration of nRBC apoptosis rates was detected in *P. vivax* patients (mean ± SD: *ex vivo*, 0.73 ± 0.31%; 24 h, 1.13 ± 0.57%) when compared with non-infected control individuals (*ex vivo*, 0.78 ± 0.27%; 24 h, 1.03 ± 0.41%) (p > 0.05; non-parametric Mann Whitney test). The same was true when *P. vivax* patients were grouped according to parasitemia density (data not shown).

This lack of erythrocytic apoptosis seems to be in consonance with the pathogenic profile of *P. vivax*. Although some reports have considered that *P. vivax* can induce severe manifestations, including acute anemia [12–14], it is classically known that infection by this parasite frequently course as a benign disease. Indeed, in contrast to *P. falciparum*, it has been believed that a simple infection by *P. vivax* is *per se* slightly competent to induce complications and that severe cases of *vivax* malaria are attributed to co-morbidities as well as high rates of *P. vivax* recurrence in areas of intense transmission as a result of drug resistance, relapse from hypnozoites or reinfection with heterologous strains [15,16]. In Brazil, where *P. vivax* is the predominant human malaria parasite and the intensity of transmission is low, severe malaria is rarely registered [9,10].

Since the classically known lower pathogenic potential of *P. vivax* [17] could be insufficient to stimulate erythrocytic apoptosis, we explored the proapoptotic effect of plasma from 10 patients with uncomplicated *P. falciparum* malaria also from Manaus, whose parasitemias are shown in Table 1, as well as from the 20 patients with *P. vivax* malaria previously studied here. A pool of plasma was tested in parallel, it was obtained from BALB/c mice either at early (4 day) or at late (7 day) stages of lethal *P. yoelii* 17XL infection [8], when mean parasitemias were, respectively, 25% and 76%, as estimated by counting the number of pRBC in thin blood smears. Plasmas from clinically healthy individuals living in Manaus and a pool of plasmas from non-infected BALB/c mice were also used as controls. Induction of apoptosis was evaluated using normal RBC (O^+^) from a clinically healthy individual living in a non-endemic malaria area. Normal RBC were incubated for 24 h and 48 h at 37°C in 5% CO_2_ in a 5% hematocrit with the plasma samples. Erythrocytic apoptosis was detected by flow cytometry using annexin V single staining, as previously described [18].

**Figure 1:**
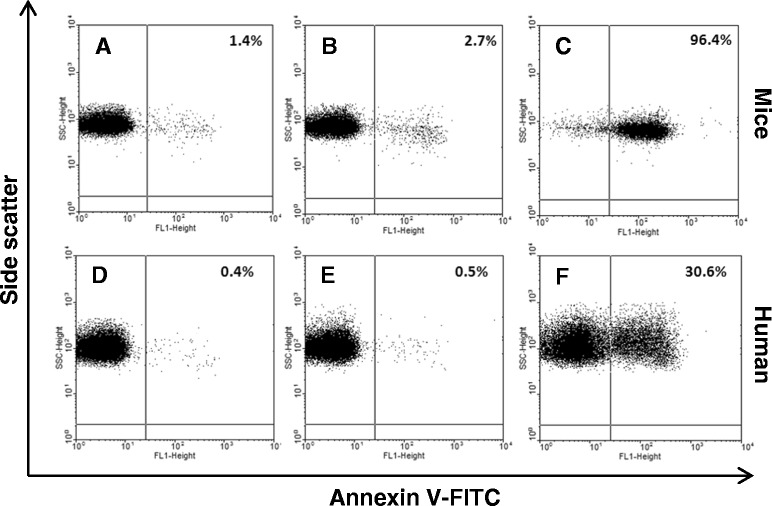
**Representative flow cytometry analysis of erythrocytic apoptosis induced by plasma.** Red blood cells (RBC) from a healthy individual were incubated for 48 h in plasma from control mice **(A)**, *P. yoelii*-infected mice (**B**, early and; **C**, late stage of infection), healthy control individuals **(D)** or malaria patients infected with *P. vivax* **(E)** or *P. falciparum* **(F)**. Apoptosis of RBC was detected using annexin V staining.

**Figure 2:**
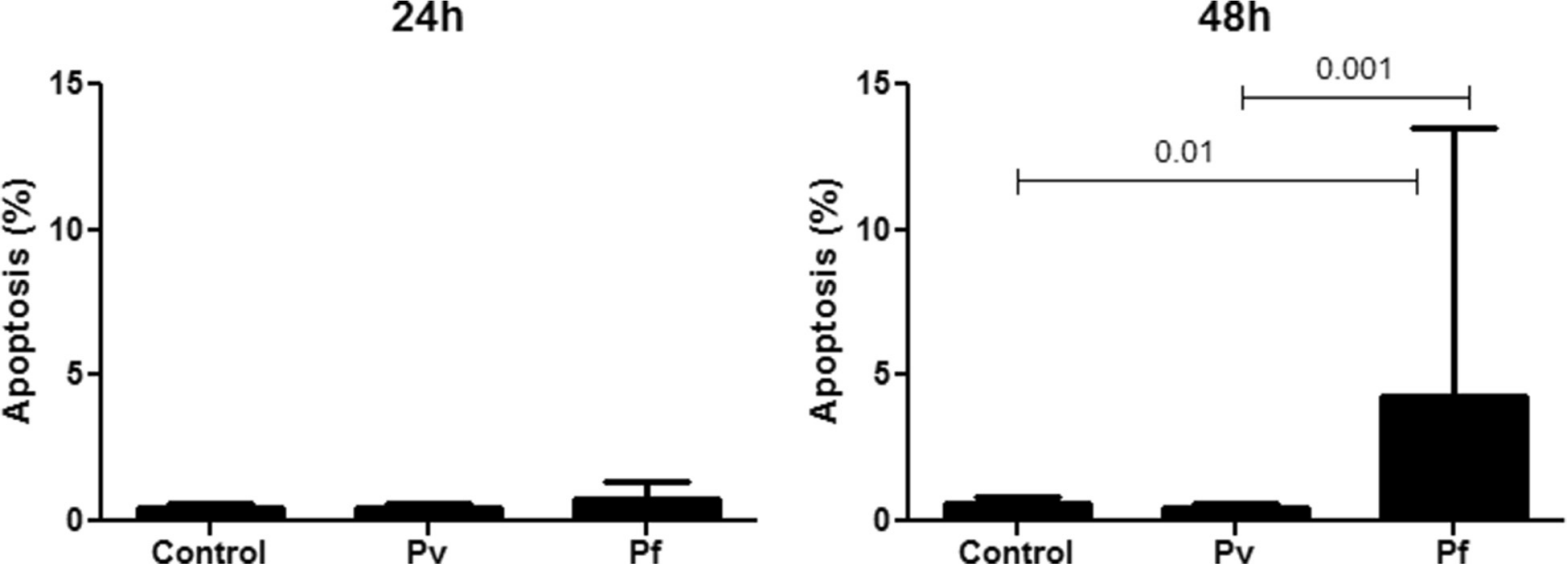
**Induction of apoptosis in normal red blood cells (RBC) by plasma from** ***P. falciparum*** **patients.** RBC from a healthy individual were incubated for 24h and 48h in the presence of plasma samples from non-infected control healthy individuals or from malaria patients infected with *P. vivax* (Pv) or *P. falciparum* (Pf). Apoptosis was detected by flow cytometry using annexin V staining. Statistical difference was tested by non-parametric Mann Whitney test.

Consistent with the data herein observed with nRBC from the *P. vivax* malarious patients, plasma from these subjects did not show any proapoptotic effects on RBC from the clinically healthy individual (Figures 1 and 2), independently of parasitemia density (data not shown). In contrast, plasma from *P. falciparum* infected patients stimulated a significant increase in the levels of apoptotic RBC after 48h of incubation (Figures 1 and 2). One of the *P. falciparum* plasmas showed an important proapoptotic effect (around 30%; Figure 1F), although no co-morbidity that could lead to the increased induction of RBC apoptosis could be diagnosed in this patient. It is remarkable that even when this patient was not considered in analysis, plasma from the *P. falciparum* patients significantly increased erythrocytic apoptosis *in vitro* (*P. falciparum vs* control, p < 0.01; *P. falciparum vs P. vivax*, p < 0.003; non-parametric Mann Whitney test). Similar to the plasma from *P. falciparum* patients, plasma from *P. yoelii* 17XL-infected BALB/c mice obtained at the stage of infection related to increased induction of nRBC apoptosis *in vivo* (late stage) [8], but not those obtained at a stage in which apoptotic nRBC was not significantly detected (early stage) [8], induced apoptosis in a high percentage (96%) of human normal RBC after 48 h of incubation (Figure 1), supporting the possibility of nRBC apoptosis induction during *P. falciparum* infection. However, it was not possible to statistically infer the relationship between parasite load and apoptotic levels during *falciparum* malaria, because the majority of *P. falciparum* patients presented similar parasitemia levels (Table 1).

If the proapoptotic properties of plasma from malarious patients do reflect the aptness of malaria parasites to induce apoptosis of normal RBC, the observation reported here seems to match the different pathogenic potentials displayed between them. In this context, it would not be surprising that the most deadly human malaria parasite, *P. falciparum* [17], stimulates higher levels of erythrocytic apoptosis that, in turn, could play a role in development of severe malaria through precocious elimination of RBC as well as occlusion of microvasculature, as suggested in studies demonstrating *in vitro* adhesiveness of apoptotic RBC to endothelial cells [19,20]. Similar phenomena related to erythrocytic apoptosis could also contribute to lethality of infection by *P. yoelli* 17XL in BALB/c mice [21], in which massive adsorption of parasite antigens on nRBC membrane due to the high parasite load seems to be the main inducer of apoptosis [8]. In the present study, however, the influence of human malaria parasitemia on erythrocytic apoptosis induction could not be accurately examined, because it was estimated by a semi-quantitative method. Nevertheless, it is known that the number of pRBC present in peripheral blood of *P. falciparum* patients does not usually reflect actual parasite load due to sequestration phenomenon of mature forms of the parasite [22]. Thus, although the majority of patients presented parasitemia that did not exceed 10,000 parasites/μL (Table 1), it is possible that parasitemia in *falciparum* patients was higher and the induction of apoptosis could be related to parasite density. Moreover, the strong proinflammatory imbalance maintained during *P. falciparum*, but not during *P. vivax* infection [23], could also contribute to erythrocytic apoptosis observed herein.

**Figure 3:**
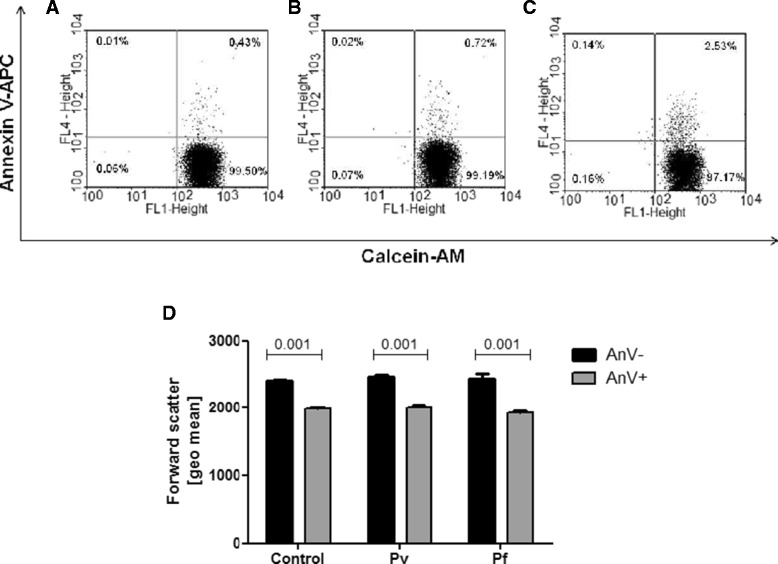
**Cell membrane integrity and cell volume in erythrocytic apoptosis induced by plasma.** RBC from a healthy individual were incubated for 48 h in the presence of plasma from non-infected control healthy individuals or malaria patients. **(A-C)** Representative flow cytometry analysis of cell membrane integrity accessed by calcein-AM and annexin V-APC double staining in RBC incubated with plasma from non-infected individuals **(A)** or from *P. vivax* **(B)** or *P. falciparum* **(C)** patients. **(D)** Forward scatter of RBC exposing (AnV+) or not (AnV-) phosphatidylserine after incubation with plasma from control non-infected individuals or patients infected by *P. vivax* (Pv) or *P. falciparum* (Pf). Statistical difference in **(D)** was tested by non-parametric Mann Whitney test.

Taking into account that labeling of PS by annexin V also occurs in non-apoptotic cells that suffered plasma membrane damage [24,25], cell membrane integrity as well as cell shrinkage were examined by flow cytometry, as additional markers of the apoptotic process, to ensure that RBC incubated with plasma were undergoing apoptosis. For this purpose, RBC from a clinically healthy individual were incubated for 48h with plasma samples (as already described) and, then, cell volume change was measured by forward scatter and cell membrane permeability was assessed using calcein-AM and annexin V-APC double staining as previously described [26]. In this way, it was possible to observe that PS-exposing RBC (AnV+) maintained cell membrane integrity and showed a decrease in cell volume, supporting that erythrocytic death process induced by plasma from *P. falciparum* patients was related to apoptosis (Figure 3).

In conclusion, the present study showed that plasma from patients with malaria caused by *P. falciparum* can induce apoptosis of normal erythrocytes *in vitro*, pointing to the possibility that this phenomenon does exist *in vivo*. This property was not recorded when plasma from patients with malaria by a less pathogenic human malaria parasite, *P. vivax* was studied. Further *ex vivo* studies are required to confirm the existence of apoptosis of nRBC during *P. falciparum* malaria to better address its clinical significance in malaria pathogenesis.

## References

[1] Bratosin D, Estaquier J, Petit F, Arnoult D, Quatannens B, Tissier JP, Slomianny C, Sartiaux C, Alonso C, Huart JJ, Montreuil J, Ameisen JC (2001). Programmed cell death in mature erythrocytes: a model for investigating death effector pathways operating in the absence of mitochondria. Cell Death Differ.

[2] Lang F, Lang E, Föller M (2012). Physiology and pathophysiology of eryptosis. Transfus Med Hemother.

[3] Wu Y, Tibrewal N, Birge RB (2006). Phosphatidylserine recognition by phagocytes: a view to a kill. Trends Cell Biol.

[4] Closse C, Dachary-Prigent J, Boisseau MR (1999). Phosphatidylserine-related adhesion of human erythrocytes to vascular endothelium. Br J Haematol.

[5] Totino PRR, Magalhães AD, Silva LA, Banic DM, Daniel-Ribeiro CT, Ferreira-da-Cruz MF (2010). Apoptosis of non-parasitized red blood cells in malaria: a putative mechanism involved in the pathogenesis of anaemia. Malar J.

[6] Koka S, Lang C, Boini KM, Bobbala H, Huber SM, Lang F (2008). Influence of chlorpromazine on eryptosis, parasitemia and survival of *Plasmodium berghei* infected mice. Cell Physiol Biochem.

[7] Eda S, Sherman IW (2002). Cytoadherence of malaria-infected red blood cells involves exposure of phosphatidylserine. Cell Physiol Biochem.

[8] Totino PRR, Pinna RA, De-Oliveira ACAX, Banic DM, Daniel-Ribeiro CT, Ferreira-da-Cruz MF (2013). Apoptosis of non-parasitised red blood cells in *Plasmodium yoelii* malaria. Mem Inst Oswaldo Cruz.

[9] Oliveira-Ferreira J, Lacerda MVG, Brasil P, Ladislau JLB, Tauil PL, Daniel-Ribeiro CT (2010). Malaria in Brazil: an overview. Malar J.

[10] Pina-Costa A, Brasil P, Di-Santi SM, Araujo MP, Suárez-Mutis MC, Santelli AC, Oliveira-Ferreira J, Lourenço-de-Oliveira R, Daniel-Ribeiro CT (2014). Malaria in Brazil: what happens outside the Amazonian endemic region. Mem Inst Oswaldo Cruz.

[11] Lança EF, Magalhães BM, Vitor-Silva S, Siqueira AM, Benzecry SG, Alexandre MA, O'Brien C, Bassat Q, Lacerda MVG (2012). Risk factors and characterization of *Plasmodium vivax*-associated admissions to pediatric intensive care units in the Brazilian Amazon. PLoS One.

[12] Tjitra E, Anstey NM, Sugiarto P, Warikar N, Kenangalem E, Karyana M, Lampah DA, Price RN (2008). Multidrug-resistant *Plasmodium vivax* associated with severe and fatal malaria: a prospective study in Papua. Indonesia PLoS Med.

[13] Manning L, Laman M, Law I, Bona C, Aipit S, Teine D, Warrell J, Rosanas-Urgell A, Lin E, Kiniboro B, Vince J, Hwaiwhanje I, Karunajeewa H, Michon P, Siba P, Mueller I, Davis TM (2011). Features and prognosis of severe malaria caused by *Plasmodium falciparum*, *Plasmodium vivax* and mixed *Plasmodium* species in Papua New Guinean children. PLoS One.

[14] Mahgoub H, Gasim GI, Musa IR, Adam I (2012). Severe *Plasmodium vivax* malaria among Sudanese children at New Halfa Hospital, Eastern Sudan. Parasit Vectors.

[15] Bassat Q, Alonso PL (2011). Defying malaria: fathoming severe *Plasmodium vivax* disease. Nat Med.

[16] Anstey NM, Douglas NM, Poespoprodjo JR, Price RN (2012). *Plasmodium vivax*: clinical spectrum, risk factors and pathogenesis. Adv Parasitol.

[17] Garcia LS (2010). Malaria. Clin Lab Med.

[18] Totino PRR, Daniel-Ribeiro CT, Ferreira-da-Cruz MF (2009). Pro-apoptotic effects of antimalarial drugs do not affect mature human erythrocytes. Acta Trop.

[19] Setty BN, Kulkarni S, Stuart MJ (2002). Role of erythrocyte phosphatidylserine in sickle red cell-endothelial adhesion. Blood.

[20] Wautier MP, Héron E, Picot J, Colin Y, Hermine O, Wautier JL (2011). Red blood cell phosphatidylserine exposure is responsible for increased erythrocyte adhesion to endothelium in central retinal vein occlusion. J Thromb Haemost.

[21] Lamb TJ, Brown DE, Potocnik AJ, Langhorne J (2006). Insights into the immunopathogenesis of malaria using mouse models. Expert Rev Mol Med.

[22] Pongponratn E, Turner GD, Day NP, Phu NH, Simpson JA, Stepniewska K, Mai NT, Viriyavejakul P, Looareesuwan S, Hien TT, Ferguson DJ, White NJ (2003). An ultrastructural study of the brain in fatal *Plasmodium falciparum* malaria. Am J Trop Med Hyg.

[23] Gonçalves RM, Scopel KKG, Bastos MS, Ferreira MU (2012). Cytokine balance in human malaria: does *Plasmodium vivax* elicit more inflammatory responses than *Plasmodium falciparum*?. PLoS One.

[24] Vermes I, Haanen C, Steffens-Nakken H, Reutelingsperger C (1995). A novel assay for apoptosis. Flow cytometric detection of phosphatidylserine expression on early apoptotic cells using fluorescein labelled Annexin V. J Immunol Methods.

[25] Lecoeur H, Prévost MC, Gougeon ML (2001). Oncosis is associated with exposure of phosphatidylserine residues on the outside layer of the plasma membrane: a reconsideration of the specificity of the annexin V/propidium iodide assay. Cytometry.

[26] Bratosin D, Mitrofan L, Palii C, Estaquier J, Montreuil J (2005). Novel fluorescence assay using calcein-AM for the determination of human erythrocyte viability and aging. Cytometry A.

